# Editorial: Recent Advances in Symbiosis Research: Integrative Approaches

**DOI:** 10.3389/fmicb.2016.01331

**Published:** 2016-08-31

**Authors:** M. Pilar Francino, Mónica Medina

**Affiliations:** ^1^Area of Genomics and Health, Fundación para el Fomento de la Investigación Sanitaria y Biomédica de la Comunitat Valenciana-Salud PúblicaValència, Spain; ^2^Unitat Mixta d’Investigació en Genòmica i Salut, FISABIO-Universitat de ValènciaValència, Spain; ^3^CIBER en Epidemiología y Salud PúblicaMadrid, Spain; ^4^Department of Biology, Pennsylvania State UniversityUniversity Park, PA, USA

**Keywords:** microbiome, multicellular host, plant-microbe symbiosis, marine symbiosis, holobiont

Symbiosis research is being transformed by new model systems and technologies that bring forth unexpected discoveries. Technological advances such as those stemming from Next Generation Sequencing enable detailed insights into the molecular bases of symbiotic relationships, and have revolutionized the study of complex microbial communities. As new data gathers, the need grows for a conceptual framework that helps organize and make sense of the information. Here, we present some ground-breaking works pushing the boundaries of our understanding of symbiosis in a variety of systems, as well as some state-of-the-art attempts at putting forward organizing principles for the whole of symbiology.

Several works in the Topic address plant-microbe symbioses. All plants are home to a variety of microorganisms that inhabit nearly all their tissues, offering a variety of benefits. Rhizosphere microbial communities are particularly critical, as they provide access to limiting nutrients. Coats and Rumpho review how molecular biodiversity analyses have advanced our understanding of the rhizosphere microbiota of invasive plants, which enables the invasion of new ranges. Coleman-Derr and Tringe, on the other hand, focus on the microbiome of crop plants and its large potential for modulating plant responses to the stresses associated with climate change and use of suboptimal agricultural lands. The relationship between the rhizosphere microbiome and stress is further demonstrated by Gehring et al. Their research on ectomycorrhizal fungal communities associated with pinetrees reveals that biotic and abiotic stressors can result in similar patterns of symbiotic community disassembly, and, remarkably, that the less diverse communities that result may actually be beneficial to host trees under stressful conditions. Kuo et al., Larrainzar et al., and Maróti and Kondorosi rather focus on the biology of specific rhizosphere microbes and the mechanisms enabling their interactions with host plants. The role of foliar symbionts is addressed by Mejía et al., who demonstrate the effects of an endophytic fungus on genetic and phenotypic expression in the tropical tree *Theobroma cacao*.

Marine systems also provide innumerable examples of fascinating symbioses. Soto and Nishiguchi offer us a new twist on the classic squid-*Vibrio* model, proposing experimental evolution approaches to gain further insights into this system. Abby et al. and Yarden explore lesser-known associations, such as those among bacteria and planktonic microalgae and among fungi and various marine invertebrates. A number of works focus on corals and their associated microbes, the study of which has been fundamental to cement the concept of the *holobiont*—a multicellular host and its associated microbiome (Margulis, 1993). Corals have become the prime example of a living system that will not survive when the interactions among the species that compose it are disrupted, as is increasingly occurring in the context of climate change. Parkinson and Baums argue that the coral holobiont should be considered as a single unit of selection, because it is specific host/symbiont genotype combinations that determine its extended phenotype and capacity for survival. Cunning and Baker remind us that the success of coral symbioses may be determined not only by the genetic identity of the partners, but also by their relative abundance, which should depend on environmental conditions. From a more mechanistic perspective, Shinzato et al. demonstrate how genomic analyses can generate detailed knowledge about the molecular and cellular processes enabling the coral-dinoflagellate symbiosis. Pernice and Levy and Roth illustrate how other technologies complement genomic analyses to generate further insight into coral physiology and metabolism. Meanwhile, Har et al. employ a variety of “omic” approaches to characterize the microbiota of *Nematostella vectensis*, a new cnidarian model for the study of metazoan evolution and development.

Insect symbioses also exemplify the varied interactions that can be established among microbes and animal hosts. Asgharian et al. revisit the many-faceted relationship between insects and *Wolbachia*, using transcriptomic techniques to show that the endosymbiont can have both sex-dependent and independent effects on its host. Hamidou Soumana et al. also exploit transcriptomics to explore the multi-level relationship among endosymbionts, trypanosomes and the tsetse fly. In turn, O'Connor et al. unveil that symbiotic microbes play a crucial role in shaping the complex interactions between the Hawaiian Drosophilidae and their host plants, hence contributing to their adaptive radiation.

In spite of their importance, host-microbe associations are vulnerable. A notorious example is the disruption of the complex microbial community inhabiting the intestinal tract when assaulted by antibiotics, resulting in an increased vulnerability to infection by opportunistic pathogens. Pérez-Cobas et al. explore how antibiotic-induced changes in the gut microbiota relate to *Clostridium difficile* infection, and define microbial taxa and functions that might protect against colonization by this pathogen. While this approach illustrates the common perspective of analyzing host-microbe symbioses in terms of potential benefits to the host, García and Gerardo take the less-traveled road of considering what microbes have to gain from the symbiotic interaction. They conclude that symbionts may sometimes be more akin to prisoners or farmed crops than to equal partners. Therefore, researchers should determine whether symbionts have adaptations to evade capture by hosts, in addition to evaluating both costs and benefits of presumed mutualisms. A theoretical framework to analyze cost/benefit ratios is provided by Hill in the context of fixed-carbon allocation in phototroph/heterotroph symbioses, where endosymbionts may control the energy trade-offs faced within host cells.

We close with two articles proposing new methodological and theoretical approaches to take forward the study of symbiotic systems. Zaneveld and Thurber demonstrate the application of ancestral state reconstruction for predicting the presence of unknown taxa and functions in microbial communities that are too complex to be wholy described experimentally. And, finally, Fitzpatrick raises the crucial issue of whether hosts and symbionts can truly coevolve when symbionts are not vertically transmitted, proposing linkage disequilibrium analysis as the correct framework to address this question. Reassuringly, this approach reveals that selection and population structure can generate covariance between host and symbiont traits, providing the basic requirement for the coevolution of the intricate symbiotic systems that pervade all realms of Life.

## Author contributions

All authors listed, have made substantial, direct, and intellectual contribution to the work, and approved it for publication.

## Funding

The authors were supported by grants NSF OCE 1442206 (MM) and MINECO SAF2012-31187 (MF) during writing of this manuscript.

### Conflict of interest statement

The authors declare that the research was conducted in the absence of any commercial or financial relationships that could be construed as a potential conflict of interest.

## References

[B1] Margulis L. (1993). Symbiosis in Cell Evolution. New York, NY: W. H. Freeman.

